# Virulence potential of faecal Escherichia coli strains isolated from healthy cows and calves on farms in Perm Krai

**DOI:** 10.18699/VJGB-22-59

**Published:** 2022-08

**Authors:** V.S. Mihailovskaya, N.B. Remezovskaya, I.N. Zhdanova, M. Starčič Erjavec, M.V. Kuznetsova

**Affiliations:** Institute of Ecology and Genetics of Microorganisms Ural Branch Russian Academy of Sciences, Perm, Russia; Institute of Ecology and Genetics of Microorganisms Ural Branch Russian Academy of Sciences, Perm, Russia; Perm Agricultural Research Institute, Lobanovo, Perm Krai, Russia; University of Ljubljana, Biotechnical Faculty, Ljubljana, Slovenia; Institute of Ecology and Genetics of Microorganisms Ural Branch Russian Academy of Sciences, Perm, Russia

**Keywords:** Escherichia coli, virulence-associated genes (VAGs), antibiotic resistance, cattle, Escherichia coli, гены, ассоциированные с вирулентностью, устойчивость к антибиотикам, крупный рогатый скот

## Abstract

Cattle are a reservoir of pathogenic and potentially pathogenic Escherichia coli (E. coli) strains, which can pose a threat to human and animal health. The aim of the study was to evaluate the occurrence of 22 virulence-associated genes (VAGs), as well as the prevalence of antimicrobial drug resistance and three different bla-genes among 49 E. coli strains isolated from healthy cattle. The presence of VAGs that are common among diarrheagenic E. coli (DEC) strains and/or extraintestinal pathogenic E. coli (ExPEC) strains was determined by amplifying specific gene sequences by PCR. The following VAGs associated with DEC were found: east1 in 24.5 % of the studied E. coli strains, estI in 10.2 %, ehxA in 8.2 %, stx2 in 6.1 %, eltA in 4.1 %, estII and stx1 in 2.0 % of the studied strains. The prevalence of ExPEC VAGs was: fimH – 91.8 %, afa/draBC – 61.2 %, iutA – 44.9 %, flu – 32.7 %, sfaDE and hlyF – 30.6 %, iroN – 22.4 %, ompT and papC – 20.4 %, kpsMTII and hlyA – 18.4 %, iss – 14.3 %, usp – 2.0 %, cnf1 and iha were not detected among the studied strains. Based on the found co-occurrence of VAGs “classical”, hetero-pathogenic and hybrid-pathogenic E. coli strains were found. E. coli strains isolated from cows had a higher diarrheagenic potential, whereas E. coli strains isolated from calves more frequently contained genes associated with the ExPEC pathotype. Among the studied E. coli strains, 77.6 % were resistant to ampicillin, 49.0 % to tetracycline, 20.4 % to chloramphenicol, 16.3 % to cefoperazone, 16.3 % to ceftriaxone, 16.3 % to aztreonam, 14.3 % to cefepime, 10.2 % to norfloxacin, 10.2 % to ciprofloxacin, 6.1 % to levofloxacin and 2.0 % to gentamicin. All strains were sensitive to meropenem and amikacin. 32.7 % of the studied E. coli strains were found to be multidrug resistant, as they were resistant to at least three groups of antibiotics. With PCR, the blaTEM, blaSHV, and blaCTX-M genes were detected in 100, 31.6, and 26.3 %, respectively, of strains resistant to at least one of the beta-lactam antibiotics. Thus, it was shown that the studied faecal E. coli of healthy cows and calves had a high hetero-pathogenic potential, therefore in the future molecular genetic characterization of these bacteria shall be an important part of the epizootic monitoring

## Abstract (ru)

Крупный рогатый скот является резервуаром патогенных и потенциально патогенных Escherichia coli (E. coli), которые могут представлять угрозу для здоровья людей и животных. Цель исследования – оценить встречаемость 22 вирулент-ассоциированных генов, а также распространенность антибиотикоустойчивости и трех генов bla различных типов среди штаммов E. coli, выделенных от здорового крупного рогатого скота. Сорок девять штаммов E. coli были проанализированы методом ПЦР на присутствие генов, распространенных среди представителей диареегенной E. coli (DEC) и внекишечной патогенной E. coli (ExPEC). Обнаружены следующие детерминанты, ассоциированные с DEC: east1 – 24.5 %, estI – 10.2 %, ehxA – 8.2 %, stx2 – 6.1 %, eltA – 4.1 %, estII и stx1 – 2.0 %. Распространенность генов ExPEC составила: fimH – 91.8 %, afa/draBC – 61.2 %, iutА – 44.9 %, flu – 32.7 %, sfaDE и hlyF – 30.6 %, iroN – 22.4 %, ompT и papC – 20.4 %, kpsMTII и hlyA – 18.4 %, iss – 14.3 %, usp – 2.0 %, cnf1 и iha не детектированы. На основании установленных комбинаций генов были определены «классические», гетеропатогенные и гибридные штаммы. Эшерихии, выделенные от коров, обладали более высоким диареегенным потенциалом, а E. coli, изолированные от телят, чаще содержали гены, ассоциированные с патотипом ExPEC. Сре- ди исследованных штаммов были устойчивыми: к ампициллину – 77.6 % культур, тетрациклину – 49.0 %, хлорамфениколу – 20.4 %, цефоперазону, цефтриаксону, азтреонаму – 16.3 %, цефепиму – 14.3 %, норфлоксацину и ципрофлоксацину – 10.2 %, левофлоксацину – 6.1 %, гентамицину – 2.0 %, все штаммы были чувствительны к меропенему и амикацину. Фенотип множественной лекарственной устойчивости имели 32.7 % культур, так как они были устойчивы к трем и более группам антибиотиков. Специфическая амплификация выявлена: к blaTEM – у 100 % штаммов, к blaSHV – у 31.6 %, к blaCTX-M – у 26.3 % штаммов, устойчивых хотя бы к одному препарату из группы бета-лактамных антибиотиков. Штаммы E. coli, изолированные от здоровых коров и телят, обладали высо- ким гетеропатогенным потенциалом, поэтому молекулярно-генетическая характеристика этих бактерий должна стать важной частью эпизоотического мониторинга.

## Introduction

Representatives of the commensal microbiota, including Escherichia coli, being obligate residents of the intestinal tract of farm animals, support physiological homeostasis and colonization resistance of the organism. At the same time, cattle, including healthy animals, present a reservoir of pathogenic and opportunistic E. coli (Chapman et al., 2006; Ewers et al., 2009; Bok et al., 2015; Madoshi et al., 2016). Diarrheagenic E. coli (DEC) causing outbreaks of intestinal diseases include various pathotypes: enteropathogenic E. coli (EPEC), enteroinvasive E. coli (EIEC), enteroaggregative E. coli (EAEC), enterotoxigenic E. coli (ETEC) and enterohemorrhagic E. coli (EHEC), which include also Shiga toxin-producing E. coli (STEC) (Allocati et al., 2013; Vila et al., 2016; Oporto et al., 2019; Santos et al., 2020).

Extraintestinal pathogenic E. coli (ExPEC) are usually divided according the infected organ system, e. g. uropathogenic E. coli (UPEC), neonatal meningitis-associated E. coli (NMEC), and sepsis-causing E. coli (SePEC). Intestinal and extraintestinal E. coli strains circulating in agricultural enterprises can pose a significant health risk to animals and humans.

Due to horizontal gene transfer, the E. coli genome is highly heterogeneous, and strains possessing genes characteristic of different pathotypes, so called hybrid-pathogenic and heteropathogenic E. coli, are known (Santos et al., 2020). Along with this, the pathogenic potential of intestinal E. coli is formed, which become sources of virulence-associated genes (VAGs) for other microorganisms, or, subsequently, themselves cause intestinal or extraintestinal infections (Chapman et al., 2006; Bélanger et al., 2011).

The widespread use of antibiotics in agriculture leads to the formation of E. coli strains with a multidrug resistance (MDR) phenotype (Pardon et al., 2017). The relationship between pathogenicity determinants and antimicrobial resistance controversial: in a number of studies, a correlation between phenotypic antibiotic resistance and the presence of certain VAGs was revealed (Suojala et al., 2010; de Verdier et al., 2012), in other studies this relationship was absent (Bok et al., 2015). In Russia, studies on the occurrence of hybrid-pathogenic and hetero-pathogenic strains of E. coli circulating among healthy animals of agricultural enterprises have not been conducted. In this regard, the analysis of the genetic profiles of pathogenicity and antibiotic resistance of E. coli strains, obligate representatives of the intestinal microbiota of cattle, is important in relation to both epizootic and epidemiological control of colibacillosis in livestock farms.

The aim of the study was to evaluate the occurrence of 22 VAGs, as well as the prevalence of antibiotic resistance and three different types of bla-genes among E. coli strains isolated from faeces of healthy cattle.

## Materials and methods

Studied strains. In the study, 49 different strains of E. coli (non-clonality of the strains was ascertained by ERIC-PCR), isolated in 2019–2021 at agricultural enterprises (n = 3) and private farms (n = 5) in Perm Krai from the faeces of cows (n = 31) and calves from 3 to 13 days of age (n = 18) were included. The strains were obtained from different animals of the Holstein black-and-white breed. The agricultural enterprises LLC “Кrasava”, LLC “Serginskoe” and LLC “Rus” specialize in dairy cattle breeding and raw milk production. The economic diet of feeding and the conditions of keeping animals (loose method) are the same and typical for these enterprises.

Detection of virulence-associated genes. To obtain matrix DNA for PCR amplification, a loop of bacterial biomass was resuspended into 100 μL of ultrapure water, heated for 15 min at 97 °C in a solid-state thermostat with a timer ТТ-2 “Termite” (Russia), centrifuged for 5 min at 13,000 rpm. The supernatants were transferred to fresh Eppendorf tubes and stored at –20 °C until usage. Twenty-two genes encoding either toxins (hlyA, hlyF, east1, ehxA, estI, estII, eltA, stx1, stx2, cnf1), adhesins (fimH, papC, sfaDE, afa/draBC, iha, flu), protectins (ompT, kpsMTII, iss), proteins of iron uptake systems (iroN, iutА) or the UPEC-specific protein (usp) were detected by PCR. Primers (LLC “Sintol”, Russia) and programs according to the recommendations of the authors (Chapman et al., 2006; Moulin-Schouleur et al., 2007) were used. Amplifications were carried out in PCR mixtures with Taq-polymerase (LLC “Sintol”) in a thermal cycler DNA Engine Dyad Thermal Cycler (Bio-Rad, USA). Band visualization and data documentation were performed using a gel documentation system Gel-DocXR (Bio-Rad).

Antimicrobial susceptibility testing. The determination of the sensitivity of E. coli strains to antibiotics was carried out in accordance with the methodical instructions MUK 4.2.1890-04 (Russia, 2004) and the clinical guidelines “Determination of the Sensitivity of Microorganisms to Antimicrobial Drugs” of the Interregional Association for Clinical Microbiology and Antimicrobial Chemotherapy (IACMAC, version-2018-03). The strains were tested by the disk-diffusion method using Muller–Hinton agar (FBIS SRCAMB, Russia) and disks (NICF, St. Petersburg, Russia) for sensitivity to penicillins (ampicillin, 10 μg), cephalosporins (cefoperazone, 75 μg; ceftriaxone, 30 μg; cefepime, 30 μg), carbapenems (meropenem, 10 μg), monobactams (aztreonam, 30 μg), aminoglycosides (amikacin, 30 μg; gentamicin 10 μg), fluoroquinolones (ciprofloxacin, 5 μg; levofloxacin, 5 μg; norfloxacin, 10 μg), tetracyclines (tetracycline, 30 μg), phenicols (chloramphenicol, 30 μg). Resistance of E. coli strains to at least one drug of three or more groups of antibiotics was defined as multidrug resistance (Magiorakos et al., 2012).

Identification of beta-lactamase genes. Detection of genes encoding TEM, SHV, and CTX-M beta-lactamase types was carried out with PCR using primers and amplification modes, according to the recommendations of the authors (Ahmed et al., 2007; Aleisa et al., 2013) with the same PCR mixtures and machines as stated above for detection of virulenceassociated genes

Statistical analysis. Qualitative features were compared using χ2 (with Yates correction) or Fisher’s exact test. Data processing was carried out using computer programs Microsoft Office XP Excel and Statistica 10.0.

## Results

Molecular characteristics of the E. coli strains

Evaluation of the prevalence of genes associated with DEC (east1, ehxA, estI, estII, eltA, stx1, stx2) and ExPEC (fimH, papC, sfaDE, afa/draBC, flu, hlyA, hlyF, ompT, kpsMTII, iss, iroN, iutА, usp) showed that they occurred with different frequencies. The iha and cnf1 genes were not detected (Table 1). All strains contained at least one VAG. The most E. coli were harbouring three (20.4 %), four (14.3 %), five (20.4 %) and six (16.3 %) genes, while the proportion of E. coli having seven or more genes did not exceed 10 %. In total, forty-five variants of VAGs combinations were identified.

**Table 1.**
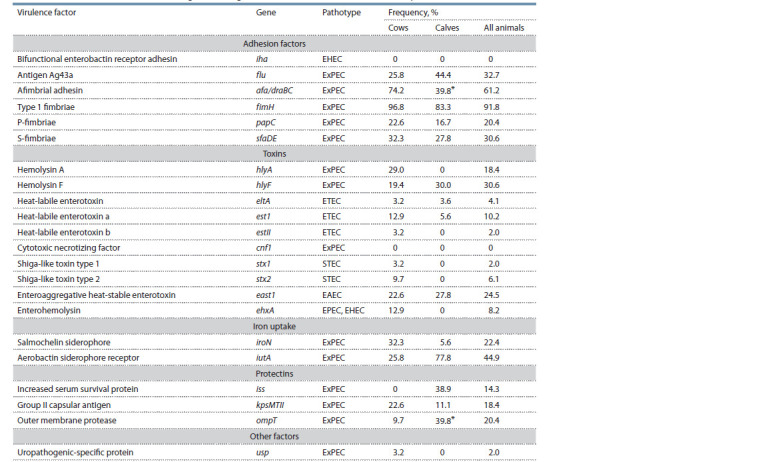
Occurrence of virulence-associated genes among E. coli strains isolated from faeces of healthy animals The difference between the samples was statistically significant, p ≤ 0.05.

**Fig. 1.**
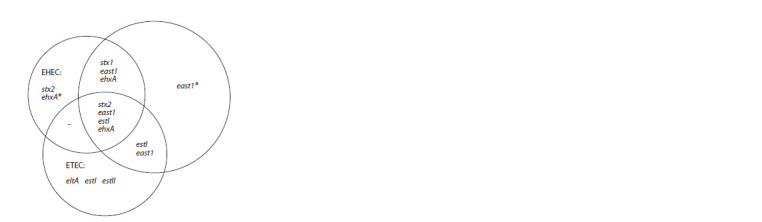
Variants of the distribution of genes associated with DEC pathotypes. The gene is associated with more than one pathotype

Prevalence of genes associated with DEC pathogenicity. Seventeen strains (34.7 %) contained genes associated with DEC pathotypes. Among the toxin-coding genes, the most common was the enteroaggregative thermostable enterotoxin east1 gene (24.5 %), which is usually, but not exclusively, associated with EAEC. Seven strains (14.3 %) carried genes associated with ETEC (estI, estII, eltA), four cultures contained STEC-marker genes stx1 (2.0 %) and stx2 (6.1 %). In four cases, ehxA was found, encoding enterohemolysin, which is the main virulence factor of EHEC, but also occurs among other diarrheal E. coli pathotypes (Jiang et al., 2015). A hetero-pathogenic strain that simultaneously contains marker genes for STEC and ETEC pathotypes was found. It should be noted that the east1 gene was detected in some E. coli strains identified as STEC and ETEC. The distribution of determinants associated with DEC pathotypes in the studied E. coli population is shown in Fig. 1.

Prevalence of genes associated with ExPEC pathogenicity. The fimH gene was the most abundant (91.8 %). The second most common gene was the afimbrial adhesin afa/draBC (61.2 %); also quite often iutA was detected (44.9 %). The pre- valence of the papC, sfaDE, flu, hlyA, hlyF, ompT, kpsMTII, iss, iroN genes varied from 14.3 to 32.7 %. Only in one case the usp gene was detected.

More than half of the strains (55.1 %) corresponded to the ExPEC group according to the classification criteria of J.R. Johnson and T.A. Russo (2005); that is, they contained two or more of the following genes: papC, sfaDE, afa/draBC, kpsMII, iutA. Interestingly, eight strains included at least three of the five genes (hlyF, iroN, ompT, iss, iutA) that were proposed by T.J. Johnson et al. (2008) to determine the APEC pathotype associated with systemic avian colibacillosis. One strain had a high uropathogenic potential because it contained the usp gene, as well as the hlyA, papC, sfaDE, afa/draBC genes often found among UPEC strains.

**Fig. 2.**
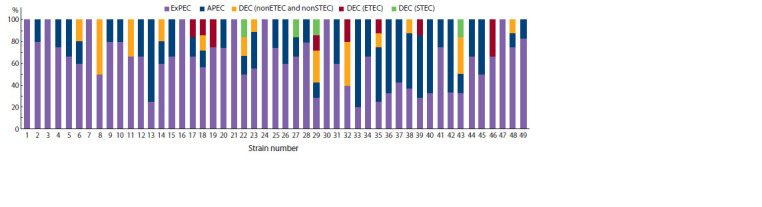
The ratio of genes associated with APEC, STEC, ETEC pathotypes, other ExPEC and DEC genes in strains isolated from healthy cows and calves.

Based on the detected combinations of genes, not only “classic” but also hybrid-pathogenic strains were identified. Eleven (22.5 %) cultures were identified that met the ExPEC criterion and included genes associated with DEC pathotypes (estI, stx2, east1, ehxA). Among them, hybrid pathotypes ExPEC/STEC and ExPEC/ETEC were found, but the prevalence of such strains did not exceed 4.1 %. The ratio of genes associated with ExPEC and DEC detected in the studied E. coli population is shown in Fig. 2.

**Fig. 3.**
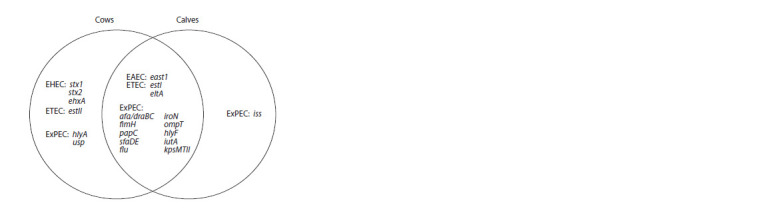
Distribution of pathogenicity determinants among strains isolated from healthy cows and calves.

Comparison of the prevalence of VAGs in subpopulations of E. coli isolated from cows and calves. Some statistical differences in the prevalence of VAGs between E. coli from samples of cows and calves were found (see Table 1). The iss gene was detected only among E. coli isolated from calves, while the stx1, stx2, ehxA, estII, hlyA and usp genes were found exclusively in E. coli isolated from cows. The ompT gene was found significantly more often in E. coli circulating among calves (p = 0.03), while the prevalence of the afa/draBC (p = 0.03) and iroN (p = 0.04) genes was higher in subpopulations of E. coli isolated from cows. In addition, the fimH, papC, sfaDE, estI, east1, kpsMTII genes were more common among the latter, but the difference was not statistically significant (Fig. 3).

Characterization of antimicrobial resistance of E. coli strains

The proportion of strains sensitive to all studied antibiotics was 12.2 %. E. coli strains resistant to only one drug were the most common in the population (36.7 %). Cultures were more often resistant to ampicillin (77.6 %) and tetracycline (49.0 %) (Table 2). It should be noted that all strains were sensitive to meropenem and amikacin.

**Table 2.**
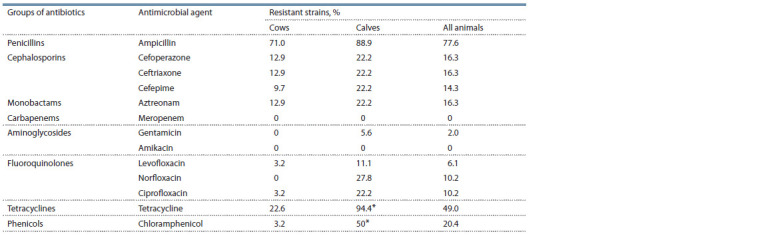
Prevalence of antibiotic resistance The difference between the samples was statistically significant, p ≤ 0.05.

Sixteen strains (32.7 %) had an MDR phenotype, while three strains were resistant to at least one antimicrobial agent from five or more groups of antibiotics. Of the fourteen identified phenotypic profiles of antibiotic resistance, seven were unique (not repeated more than once). The most common were strains with the phenotype of resistance to ampicillin (32.7 %), ampicillin and tetracycline (12.3 %), as well as ampicillin, tetracycline and chloramphenicol (10.2 %).

Prevalence of beta-lactam resistance genes. Thirty-eight E. coli strains (77.6 %) were resistant to at least one betalactam antibiotic. These strains were tested for the presence of beta-lactamase genes. Specific amplification for blaTEM was detected in 100 % of cases, for blaSHV – 31.6 %, for blaCTX-M – 26.3 %.

Comparative analysis of the prevalence of drug resistance in subpopulations of E. coli isolated from cows and calves. It should be noted that strains resistant to gentamicin and norfloxacin were found only among E. coli obtained from calves. In the same group, the occurrence of E. coli representatives that were not sensitive to tetracycline and chloramphenicol, as well as those with the MDR phenotype, was significantly higher (p < 0.01). The proportion of strains resistant to other antimicrobial agents was also higher in the calf group, although the differences were not statistically significant (see Table 2).

Relationship between virulence factors and antimicrobial resistance

In the group of strains with the MDR phenotype, E. coli containing five or more VAGs were found more often (p = 0.04), and the probability of finding the hlyA, iss, iutA genes in this group was higher than among E. coli without the MDR phenotype (p ≤ 0.05). In the group of strains in which five or more pathogenicity genes were detected, the proportion of E. coli resistant to five or more antimicrobial agents was significantly higher (p = 0.04). It should be noted that among E. coli with the MDR phenotype, there were E. coli containing the marker genes estI, eltA (ETEC), stx1 (STEC), as well as six strains identified as APEC.

## Discussion

E. coli strains circulating in agricultural settings can pose a significant risk to human health (Bélanger et al., 2011; Manges et al., 2016). On the one hand, the possibility of transmission of pathogenic E. coli through food products, including cattle meat, has been revealed (Vincent et al., 2010). On the other hand, the presence of similar phylogroups, serotypes and genetic determinants of pathogenicity in representatives of E. coli that cause human diseases and E. coli of animal origin suggests that animals can be a reservoir of opportunistic E. coli, as well as pathogens of zoonotic infections (Tivendale et al., 2010; Mora et al., 2013). For example, farm animals are the main natural reservoir and source of STEC strains that cause hemorrhagic colitis in humans (Onishchenko et al., 2015).

The presence of certain virulence factors in the pathogen causes the manifestation of clinical symptoms of intestinal and extraintestinal infections caused by E. coli, the corresponding pathological groups – DEC and ExPEC (Chapman et al., 2006; Dale, Woodford, 2015). According to numerous studies, these strains can circulate among the microbiota of healthy animals that do not have pronounced symptoms of the disease, in addition, some VAGs may be present in the genomes of commensal E. coli (Orden et al., 2002; Ewers et al., 2009, 2021; Bok et al., 2015). Our studies showed that E. coli strains isolated from healthy cattle were characterized by a high level of genetic diversity and contained pathogenicity determinants associated with pathotypes DEC and ExPEC. ExPEC strains were the most common, as they were found in 55.1 % of the studied strains. E. coli containing marker genes of diarrheagenic pathotypes: STEC (in 8.1 % of cases) and ETEC (14.3 %) were also detected. Similar data were presented in the study by J.A. Orden et al. – among the strains obtained from healthy cattle, there were representatives of STEC and EPEC with frequencies of 8.7 and 8.2 %, respectively (Orden et al., 2002), whereas the prevalence of ETEC and STEC representatives isolated from dairy cows in China was only 4.29 and 1.98 % (Huasai et al., 2012). It should be noted that in our sample, individual VAGs were detected with a high frequency (fimH – 91.8 %; afa/draBC – 61.2 %; iutA – 44.9 %; sfaDE – 30.6 %). R.V. Pereira et al. (2011) found that the fimH and iutA genes were more prevalent among E. coli isolated from healthy calves – in 100 and 86.9 % of cases respectively, while the sfaDE and afa/draBC genes were found less frequently – in 4.9 and 1.6 % of cases, respectively.

When comparing the prevalence of pathogenicity determinants in strains circulating among healthy cattle of Russian and Slovenian farms, it was found that faecal E. coli strains from Slovenian cows had a lower virulence potential, since the occurrence of VAGs was significantly lower: fimH – 65.2 %, hlyA – 9.0 %, stx2, ompT and kpsMT – 3.4 %, usp – 1.1 %, and the sfaDE, iroN, cnf1 genes were not detected at all (data not shown).

Recently, more researchers have noted that VAGs associated with either ExPEC or DEC are found among atypical E. coli pathotypes (Santos et al., 2020; Ewers et al., 2021). Such strains can cause severe infectious diseases in both farm animals and humans. In 2011, an outbreak of food poisoning was recorded in Germany, caused by a hetero-pathogenic strain of E. coli O104 : H4 with a rare combination of VAGs (stx2 and aatA, aggR, aar, aggA, aggC), characteristic of two different groups of diarrheagenic E. coli – STEC and EAEC (Bielaszewska et al., 2011). It was reported that hetero-pathogenic strains can be isolated from animals and food (Cheng et al., 2006; Monday et al., 2006).

In our study, strains were found that included the stx1, stx2 genes and the gene of enteroaggregative thermostable enterotoxin east1, which is often found in EAEC strains. However, to determine this pathotype, it is necessary to identify additional determinants, and also to perform phenotypic studies (Boisen et al., 2020). ExPEC/STEC hybrids are also high-risk pathogens because they cause both diarrhoea and extraintestinal infection. We found hybrid-pathogenic and hetero-pathogenic strains in 2.0 and 4.1 % of cases, respectively.

Our study revealed that the VAG profiles of E. coli strains circulating among healthy cows and calves had specific differences. The occurrence of VAGs (except for ompT, hlyF, iutA) was higher among E. coli isolated from cows; moreover, genes stx1, stx2, ehxA and estII associated with DEC were detected exclusively in this sample. Interestingly, among E. coli isolated from calves, the genes ompT, hlyF, iutA, iss were detected more often. Thus, E. coli living in the intestines of healthy cows had a high diarrheagenic potential, while ExPEC genes were common in both samples; however, in the group of calves, E. coli containing genes associated with the APEC pathotype were more common. Perhaps these differences are related to the fact that bacteria of the DEC pathogroup can persist in the intestines of cows without causing active infection, since the “mature” microbiome provides colonization resistance, while calves are more vulnerable to DEC, which often cause diarrhoea and death of young animals in the first days of life (Bashahun, Amina, 2017). In addition, natural immunity formed in previously ill adult animals, as well as post-vaccination immunity, provide tolerance to most pathogenic E. coli.

Agriculture accounts for up to 70 % of antimicrobial drug consumption, so productive animals are the main arena for the emergence of bacterial antibiotic resistance and the emergence of strains with multiple drug resistance (Berge et al., 2009; Pereira et al., 2011; Okello et al., 2021). It was shown that among E. coli isolates circulating in poultry and agricultural enterprises, more than half had the MDR phenotype1.

1 Zabrovskaya A.V. Epizootological analysis of the spread of antibioticresistant strains of pathogens of infectious diseases of farm animals in the North-Western federal district of the Russian Federation: Doctor Sci. (Vet.) Dissertation. St. Petersburg, 2019. 323 p.

Significant differences in the prevalence of antibiotic-resistant microorganisms circulating in livestock farms in different countries may be due to the peculiarities of animal housing conditions and the use of antimicrobial drugs. This determines the expediency of a comparative study of transmission routes and mechanisms of acquiring antibiotic resistance.

Beta-lactam antibiotics and tetracycline preparations are most widely used in veterinary medicine for treatment and prevention of infectious diseases of cattle (Berge et al., 2009; Pereira et al., 2011). Of particular importance is the growing resistance of microorganisms to extended-spectrum cephalosporins (third and fourth generation), as these antibiotics are critically important for medicine2. 2.Antibiotics in Animal Farming. Public Health and Animal Welfare, 2011.According to our study, strains with the MDR phenotype isolated from healthy cows and calves were found with a high frequency (32.7 %). In the study sample, 77.6 % of cultures were resistant to at least one antimicrobial agent of the beta-lactam group of antibiotics (16.3 % – to cefoperazone and ceftriaxone), 49.0 % – to tetracycline, and 20.4 % – to chloramphenicol. These data significantly exceed the values published by B.P. Madoshi et al. (2016), who found the proportion of strains isolated from healthy cattle and resistant to ampicillin, tetracycline and chloramphenicol was 21.3, 33.1 and 4.4 %, respectively. Only 3.7 % of the strains were resistant to cefotaxime (Madoshi et al., 2016). Even lower resistance to cephalosporins (1.5 %) was demonstrated by E. coli strains isolated from cattle faeces at agricultural enterprises in Japan (Sato et al., 2014).

Beta-lactamase production is one of the main mechanisms of resistance to beta-lactam antibiotics. In the studied strains resistant to at least one agent from this group of antimicrobial drugs, genes and combinations of beta-lactamase genes of the TEM, SHV and CTX-M families were found. This fact may be related to the widespread use of beta-lactam antibiotics in enterprises of Perm Krai. However, it was found that even among strains isolated from cattle on farms where antibiotics were rarely used, the occurrence of blaCTX-M ranged from 2.3 to 25.0 % (Lee et al., 2020). Attention should be paid to the high occurrence in E. coli strains of genes encoding betalactamases, the plasmid localization of which can contribute to the effective spread of antibiotic resistance within the microbial population through horizontal transfer.

According to our data, in general, resistance to antimicrobial agents was more common in the E. coli subpopulation isolated from calves than among E. coli isolated from adult animals. The largest differences were observed for tetracycline (94.4 versus 22.6 %) and chloramphenicol (50.0 versus 3.2 % resistant strains from calves and cows, respectively). Perhaps this is due to the addition of these drugs to the calves’ feed for a long period, since it is known that antibiotics are often added to milk or milk substitutes in order to prevent diseases and treat diarrhoea, which is the main cause of mortality of calves before weaning (Berge et al., 2009; de Campos et al., 2021; Okello et al., 2021).

It is known that the phenotype of resistance of bacteria circulating among calves is mainly a consequence of the use of antibiotics in enterprises (DeFrancesco et al., 2004; Sato et al., 2005). Antibiotics of the aminoglycoside group – neomycin and gentamicin, are of great importance for the prevention and treatment of streptococcal and staphylococcal infections in calves3. 3 Esaulenko N.N. The effectiveness of the use of the probiotic “Sporothermin” in the diets for heifers: Cand. Sci. (Agric.) Dissertation. Krasnodar, 2015. 118 p.This may explain that E. coli strains resistant to gentamicin and norfloxacin were found only among E. coli derived from calves.

## Conclusion

Microbiological monitoring of pathogenic and conditionally pathogenic microorganisms isolated from farm animals and from animal products is currently carried out at all enterprises of the Russian Federation. This monitoring is important, as bacteria in the herd can circulate between animals of all ages over a long period of time, posing a risk to the animals themselves and to the personnel.

This paper presents for the first data on the prevalence of VAGs, as well as the occurrence of hybrid-pathogenic and hetero-pathogenic strains of E. coli circulating among healthy animals at agricultural enterprises in the European part of Russia (Perm Krai). In addition, the relationship between the virulence potential of E. coli and their antibiotic resistance was analysed. Another important aspect presented in the work is a comparative analysis of the biological properties of E. coli strains isolated from different age groups of animals – cows and calves.

Studies have shown that E. coli strains circulating among healthy animals on farms and agricultural enterprises were characterized by a high hetero-pathogenic potential. In the E. coli population under consideration, representatives of DEC (including STEC and ETEC), which can cause intestinal infections, as well as ExPEC, causing extraintestinal infections, were common. In addition, hybrid strains combining genes associated with different E. coli pathotypes were found. Strains with the MDR phenotype had a high virulence potential, since they more often contained more than five VAGs. E. coli isolated from cows showed a higher diarrheagenic potential, while E. coli isolated from calves more often contained genes associated with the ExPEC pathotype. E. coli obtained from calves generally showed greater resistance to antimicrobial agents than E. coli isolated from adult animals.

The obtained data on the molecular properties of microorganisms of the intestinal microbiota of healthy cattle allow to assess their epizootic significance and can serve as a basis for the formation of a monitoring system for colibacillosis in agricultural enterprises.

## Conflict of interest

The authors declare no conflict of interest.
